# Protein-Based Vaccine Protect Against *Piscirickettsia salmonis* in Atlantic Salmon (*Salmo salar*)

**DOI:** 10.3389/fimmu.2021.602689

**Published:** 2021-02-17

**Authors:** Juan Pablo Pontigo, Carla Espinoza, Mauricio Hernandez, Guillermo Nourdin, Cristian Oliver, Rubén Avendaño-Herrera, Jaime Figueroa, Cecilia Rauch, José M. Troncoso, Luis Vargas-Chacoff, Alejandro J. Yáñez

**Affiliations:** ^1^Laboratorio de Biotecnología Aplicada, Facultad de Medicina Veterianaria, Universidad San Sebastián, Puerto Montt, Chile; ^2^Instituto de Bioquímica y Microbiología, Facultad de Ciencias, Universidad Austral de Chile, Valdivia, Chile; ^3^Proteogenomics Laboratory, Molecular Epidemiology for Life of Science reseArch (MELISA) Institute, San Pedro de Paz, Chile; ^4^Laboratorio de Biotecnología Acuática, Facultad de Ciencias Veterinarias. Universidad Austral de Chile, Valdivia, Chile; ^5^Interdisciplinary Center for Aquaculture Research (INCAR), Concepción, Chile; ^6^Laboratorio de Patología de Organismos Acuaticos y Biotecnologia Acuicola, Facultad de Ciencias Biologicas, Universidad Andres Bello, Viña del Mar, Chile; ^7^Cargill Innovation Center, Calbuco, Chile; ^8^Instituto de Ciencias Marinas y Limnológicas, Universidad Austral de Chile, Valdivia, Chile; ^9^Centro Fondap de Investigación de Altas Latitudes (IDEAL), Universidad Austral de Chile, Valdivia, Chile; ^10^Facultad de Ciencias, Universidad Austral de Chile, Valdivia, Chile

**Keywords:** *Piscirickettsia salmonis*, Atlantic salmon, innate immunity, vaccine, proteome, IgM, IL-1b

## Abstract

An effective and economical vaccine against the *Piscirickettsia salmonis* pathogen is needed for sustainable salmon farming and to reduce disease-related economic losses. Consequently, the aquaculture industry urgently needs to investigate efficient prophylactic measures. Three protein-based vaccine prototypes against *Piscirickettsia salmonis* were prepared from a highly pathogenic Chilean isolate. Only one vaccine effectively protected Atlantic salmon (*Salmo salar*), in correlation with the induction of Piscirickettsia-specific IgM antibodies and a high induction of transcripts encoding pro-inflammatory cytokines (i.e., Il-1β and TNF-α). In addition, we studied the proteome fraction protein of *P. salmonis* strain Austral-005 using multidimensional protein identification technology. The analyzes identified 87 proteins of different subcellular origins, such as the cytoplasmic and membrane compartment, where many of them have virulence functions. The other two prototypes activated only the innate immune responses, but did not protect *Salmo salar* against *P. salmonis*. These results suggest that the knowledge of the formulation of vaccines based on *P. salmonis* proteins is useful as an effective therapy, this demonstrates the importance of the different research tools to improve the study of the different immune responses, resistance to diseases in the Atlantic salmon. We suggest that this vaccine can help prevent widespread infection by *P. salmonis*, in addition to being able to be used as a booster after a primary vaccine to maintain high levels of circulating protective antibodies, greatly helping to reduce the economic losses caused by the pathogen.

## Introduction

The aquaculture industry is constantly under threat from infectious diseases, and although antibiotics and chemical treatments have proven to be useful, they present major environmental and economic concerns. Therefore, immunoprophylactic therapies, such as vaccines, need to be developed and applied to prevent and control disease (1, 2). Vaccination has become one of the most important prophylactic tools for disease control in modern industrial aquaculture (3).

*Piscirickettsia salmonis* is the etiological agent of salmonid rickettsial septicaemia (SRS) or *Piscirickettsiosis*. This bacterium was first isolated from Coho salmon, and the piscirickettsial organism recognized as a fish pathogen in 1989, was designated the LF-89 type strain (4–6). *P. salmonis* has been confirmed as the agent responsible for this disease in various salmonids grown on the Atlantic and Pacific coasts of the United States and Canada, as well as in Ireland, Norway, Scotland, and Tasmania (4, 7–10). In Chile, SRS primarily affects cultured salmonids, such as Coho salmon (*Oncorhynchus kisutch*), Atlantic salmon (*Salmo salar*), and rainbow trout (*Oncorhynchus mykiss*) (11). In addition, *P. salmonis* has been confirmed as a pathogen of other species, such as European sea bass [*Dicentrarchus labrax* (L.)] in Greece (12, 13) and the white sea bass [*Atractoscion nobilis* (Ayres)] in the United States (14, 15).

*P. salmonis* is a Gram-negative, non-motile, non-encapsulated, facultative intracellular, pleomorphic bacterium with a predominantly coccoid shape measuring 0.5–1.5 μm in diameter. Molecular phylogenetic analyses, based on 16S rRNA gene sequencing, have categorized *P. salmonis* as a γ-proteobacteria in the *Piscirickettsiae* class, with relationship to *Coxiella, Francisella*, and *Legionella* (4). This bacterium produces a systemic infection characterized by colonization of the kidney, liver, spleen, intestine, brain, ovary, and gills. However, the mechanisms of bacterial virulence and pathogenesis remain poorly understood (6, 16). *Piscirickettsia salmonis*, being an intracellular bacterium, has been described as using macrophages as an infection strategy, in addition to replicating in cytoplasmic vacuoles as a mechanism for evasion of the host's innate immunity (17). Currently, antimicrobial agents are ineffective, and the high mortality rate causes annual losses in excess of US$700 million in Chile (7, 17, 18).

In the Chilean salmon industry, the management strategy has focused primarily on SRS control through vaccination and antimicrobial therapies. While antibiotics can prophylactically inhibit pathogen growth, these have had little success in stopping new disease outbreaks (19). Furthermore, the use of antimicrobials in the Chilean aquaculture industry has steadily increased in correlation with intensified salmonid production. Related to this, data from the National Fisheries and Aquaculture Service of Chile (SERNAPESCA) confirm that Atlantic salmon cultures receive the largest amount of antibiotics, with respect to the other species in cultivation. Of this amount, *Piscirickettsiosis* garners the most attention, with a 98.3% total of antibiotics administered in the control of SRS mainly oxytetracycline and florfenicol in seawater (20). However, reduced sensitivity to florfenicol and oxytetracycline has been reported in salmon farms, in addition to increased resistances to other antibiotics such as penicillin, streptomycin, oxolinic acid, and oxytetracycline (20).

The lack of effective control treatments for SRS highlights the need for different options, such as new, non-bacterin types of vaccines. Vaccines based on inactivated bacteria can successfully control diseases (2), but currently existing preparations based on *P. salmonis* provide low or variable protection against SRS (6, 7, 21). Outcome differences may be related to variations in epitopes caused by inactivation treatments. Furthermore, different vaccination protocols are intensively used by the Chilean salmon industry, including whole bacterium, inactivated, and adjuvanted vaccines for primary intraperitoneal immunization, which in some cases can be followed by an oral boost (22). However, the efficacy of each of the vaccine formulations is not completely effective, mainly due to the contradictory results obtained with protocols based on bacterins, in addition to the complete ignorance of whether vaccination will grant humoral immunity, most of the time opsonized by professional phagocytes, without obtaining the desired effect of long-term protection (22).

This current study analyzes the effectivity in Atlantic salmon of three SRS vaccine formulations based on proteins isolated from *P. salmonis*. All formulation induced an innate immune response, however, protection of fish against a lethal-dose challenge of *P. salmonis* occurred only when high levels of IgM were produced. This protection was only induced with one of the formulations against *P. salmonis*, this observation suggests that one of the most important issues for induction is a high amount of anti-*P. salmonis* specific IgM. In addition, during this study it was possible to characterize some proteins involved in virulence through the multidimensional protein identification technology (MudPIT) found from one of the formulations of the vaccine from the Austral-SRS 005 strain.

In summary, this study is an approach to the complexities that are present in the host-pathogen interaction, related to the immune response and various characterized virulence factors, observing that diverse integral technologies for the development of effective vaccines against *Piscirickettsia salmonis*.

## Materials and Methods

### Fish Maintenance

**Figure 1:**
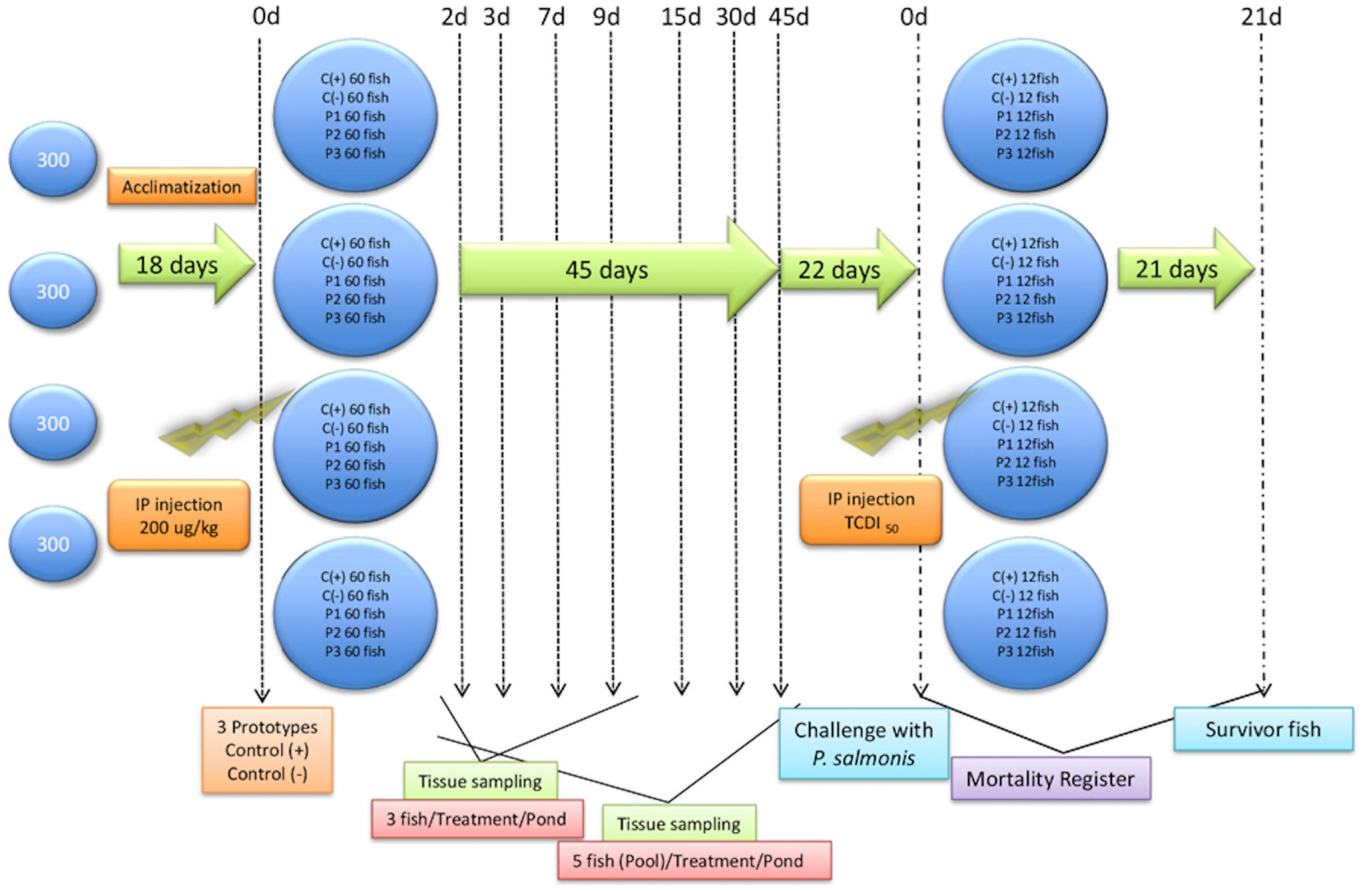
General trial outline. General trial outline showing fish entry, acclimatization days, injection formulations, and sampling days. Prototype 1 (P1), Prototype 2 (P2), and Prototype 3 (P3); Positive controls (C+) commercial *P. salmonis* vaccine, and negative controls (C-) used PBS mixed with a non-mineral oil adjuvant.

First, 1,260 Atlantic salmon (*Salmo salar*, 35 ± 3,8 g) were obtained from the Los Fiordos salmon farm and subjected to health check analyses, using accredited laboratories, to verify pathogen free status. All subsequent bioassays were conducted at the Quillaipe Experimental Station (Fundación Chile). Animals were divided into five tanks (1 m^3^), four of which contained 300 fish per tank and one of which held 60 reserve fish in case of mortalities (Figure 1). All fish were fed *ad-libitum* for 24 h with the commercial Transfer 50® (EWOS) diet. The experiment was reviewed by an internal animal welfare committee of the Foundation of Chile (PPT256-01). In addition, the study adhered to animal welfare procedures and was approved by the bioethical committees of the Universidad Austral de Chile and the National Commission for Scientific and Technological Research (ANID) of the Chilean government.

### Production of *Piscirickettsia salmonis* Protein

**Figure 2:**
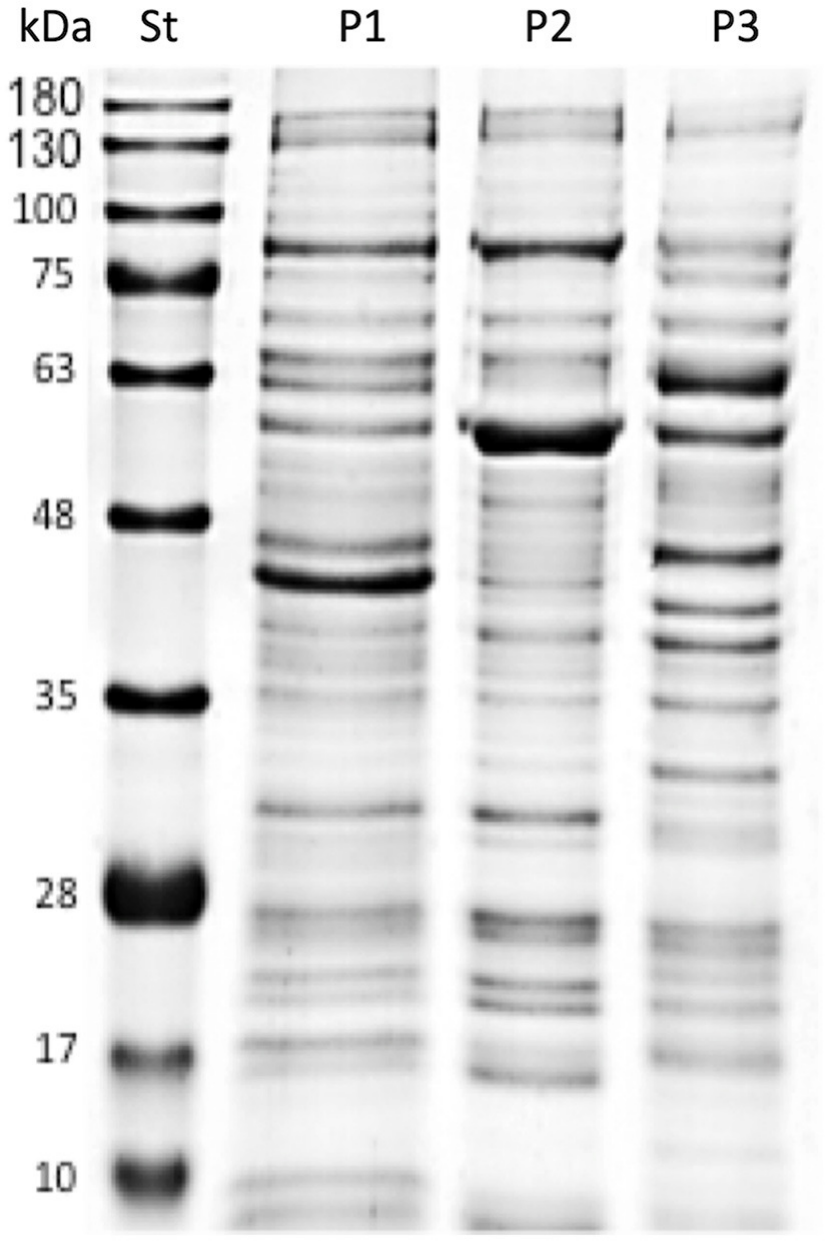
SDS-PAGE analysis of protein *P. salmonis* vaccine formulations. Standard molecular weight markers (St), Prototype 1 (P1), Prototype 2 (P2), and Prototype 3 (P3) indicate the different protein profiles of the *P. salmonis* vaccine prototypes.

Bacterial suspensions of AUSTRAL-005 strain were prepared in sterile Austral-broth (10), and 15 mL aliquots were adjusted to 1.0 absorbance at 600 nm, as measured in a spectrophotometer. The aliquots were washed with 1 × phosphate buffered saline [(PBS), pH buffer] by centrifugation at 6,000 × *g* for 5 min at 4°C, according to the protocol of Oliver et al. and Yañez et al. (23, 24). The samples were further centrifuged at 13,000 × *g* for 10 min at 4°C In presences of protease inhibitor to obtain the supernatant (24), the samples were subsequently disrupted at 80 W for intervals of 20 s, for 2 min. Centrifuge at 5,500 × *g* at 4° C collecting the supernatant (proteins for the vaccine prototype one formulation). The supernatant is ultracentrifuged at 40,000 × *g* for 2 h at 4°C, the pellet corresponds to Prototype 2 (P2) and the supernatant to Prototype 3 (P3). Later it was carried out for an SDS-PAGE electrophoretic mobility assay (Figure 2).

### Fish Sampling

Three hundred fish from each tank were anesthetized with Here pond-S® (17 mg/L). After 5 min, the passive integrated transponder (PIT) tags were recorded using a magnetic reader (Trovan®). Blood samples (500 μL) were taken from the tail vein, left to clot for 30 min at room temperature, and centrifuged (1,500 × *g* for 5 min) to obtain 100 μL of serum. The fish were pre-bleed previously. Following blood sampling, head kidney and liver samples were frozen in liquid nitrogen and stored at −80°C.

### Vaccination Protocols

Three experimental formulations of different highly immunogenic protein fractions [Prototype 1 (P1), Prototype 2 (P2), and Prototype 3 (P3)] from *Piscirickettsia salmonis* were prepared. Each protein mixtures were emulsified with one volume Montanide^TM^ ISA 763 AVG® adjuvant to obtain a 1:1 oil-in-PBS preparation. Three groups of fish were injected intraperitoneally (IP) with 0.1 mL of each vaccine preparation containing 200 μg/kg of each *P. salmonis* protein fraction. For positive controls (C+) commercial, broad spectrum *P. salmonis* vaccine, while negative controls (C-) used PBS mixed with adjuvant (Montanide^TM^ ISA VG® 763 A). Vaccinated fish were fed *ad-libitum*, moved to a room with four bio-secure tanks (1 m^3^), distributed at a density of 60 fish/tank, and kept in seawater previously filtered and treated with ultraviolet.

A lethal dose (LD50) of bacteria was calculated, and fish were challenged with 0.1 mL of 10^8.5^ TCID_50_/mL of live bacteria. Serial dilutions were prepared from semi-purified *P. salmonis*. The control group (C-) was injected with saline solution. Injected fish from each group were distributed into two tanks and maintained at 14°C under controlled conditions. Mortalities were recorded every 12 h for 21 d to determine the relative percent survival (RPS) and define vaccine efficiencies and powers (Figure 1).

$$\text{RPS}=\left(1-\frac{\text{\%Vaccinated fish mortality}}{\text{\%Control fish mortality}}\right)\times 100$$

Additionally, visual inspection of all fish and necropsies were performed after any mortality since lesions would indicate if a fish died due to *P. salmonis*. SRS was confirmed through histopathological and PCR analyses (25) of head kidney and liver samples taken from dead fish (data not shown).

### ELISA

The ELISA assay was performed as described by Birkbeck et al. and Sotomayor-Garding et al. (26, 27) with same modifications. Firstly, MaxiSorp® (Nunc™ 439454) ELISA plates were incubated overnight at 4°C with 100 μL/well of total proteins mixed fraction of *P. salmonis* antigens in PBS, containing proportional amount of P1, P2, and P3 (1.0 μg/μL). The internal positive plasma control used for the normalization of the ELISA readings on the plates was produced from the pre-bleeding of the fish prior to the challenge tests. Absorbed antigens were blocked with 100 μL of blocking solution [1% bovine serum albumin (BSA)-PBS] for 2 h at 20°C. After washing with 100 μL of wash solution (PBS- 0.05% Tween 20), 100 μL of 1/50 PBS-diluted *S. salar* serum was added and incubated for 1 h at 20°C. The plates were washed and incubated with monoclonal mouse anti-salmon IgM, isotype IgG1 (BiosChile, IGSA, Chile) for 1 h at 20°C. The plates were washed again and incubated with horseradish peroxidase-conjugated goat anti-mouse IgG (Santa Cruz Biotechnology). Serum antibody titers were determined using 3,3,5,5-tetramethylbenzidine (Sigma-Aldrich) as a chromogenic substrate, with H_2_SO_4_ used to stop the reaction. Values were obtained by measuring absorbance at 450 nm.

### Gene Expression Analysis

Head kidney samples were used to assess changes in IL-1β and TNF-α expression. Total RNA was extracted with the TRIzol reagent (Invitrogen) following the manufacturer's instructions, and the obtained samples were treated with amplification grade DNase I (1 U μg^1^ RNA, Invitrogen). The SuperScript III RNase H ReverseTranscriptase platform (Invitrogen) synthesized first-strand cDNA from 1 μg of total RNA using the oligo-dT18-22 primer at 50°C for 60 min. Total cDNA was then used as a template for real-time PCR reactions using 7.5 μL Brillant SYBR® Green II (qPCR Master Mix, Stratagene®) and 50 nM of specific primers (for genes IL-1β, TNF-α), and 1 μL of each template (1:10 dilution, in triplicate) in a total volume of 15 μL. The following reaction conditions were used for the Stratagene p3000X® real-time PCR thermocycler: 95°C for 30 s, 58°C for 30 s, and 72°C for 15 s in 45 cycles. The mRNA gene expressions were normalized to the Atlantic salmon β-actin using the comparative ΔΔCt method (28). The oligonucleotides used were: IL-1β: forward *5*′*-ccacctgctcaacttgc-3*′ and reverse *5*′*-gcagctccatagcctcactc-3*′; TNF-α: forward 5′-*cgtggtgtcagcatggaaga*-3′ and reverse *5*′*-agtatctccagttgaggctccatt-3*′; y β-actin (housekeeping gene): forward 5′-*gacaacgcatccggtatgtgc-3*′ and reverse *5*′*-cagctcgttgtagaaggtg-3*′ and 18s (housekeeping gene) described by Pontigo et al. (29). In all cases, each qPCR was performed with triplicate samples and repeated with at least two independent samples.

### Sample Preparation for Proteomics Analysis

AUSTRAL-005 strain were incubated in lysis buffer (50 mM Tris–HCl, pH 7.5; 150 mM NaCl; 1% NP-40; 0.5% sodium deoxycholate; and 1% SDS) for 1 h at 4°C. Finally, the solution was sonicated for10 min at 4°C at a frequency of 20 kHz, lyophilized and stored at −20°C until use. All samples were analyzed by SDS-PAGE.

The proteins of P1 prototypes were subjected to precipitation using 5: 1 v / v cold acetone 100% v/v and incubated overnight at −20°C, then they were centrifuged at 15,000 × *g* for 10 min, the supernatant was discarded, and the pellet was washed three times with acetone at 90% v / v, later the proteins were dried in a rotary concentrator at 4°C, and finally they were resuspended in 8 M urea with 25 mM of ammonium bicarbonate pH 8. Subsequently, proteins were reduced at room temperature for 30 min with 2 mM dithiothreitol and alkylated in the dark at room temperature for 30 min with 10 mM iodoacetamide. The reaction was diluted eight times with 25 mM NH_4_HCO_3_, pH 7.5; 2 μL of 0.1 ng/mL modified trypsin (Promega, Madison, WI, USA) was added, and the reaction was incubated at 37°C for 16 h. The reaction was stopped by adding acetic acid, pH 2.0.

### Protein Identification by MudPIT

Tryptic peptides were concentrated on a CentriVap Concentrator (Labconco, USA) to a final volume of 20 μL and loaded on a 350 μm ID fused silica 2D high-performance liquid chromatography triphasic peptide trap column packed *in-house* with 3 cm of a reverse-phase desalting C18 (100 Å, 5 μm Magic C18 particles; Michrom Bioresources, Auburn, CA,USA), 3 cm of a strong cation exchange column (300 Å, 5 μm, PolySULFOETHYL A; PolyLC Inc., Columbia, MD, USA), and, finally, 3 cm of reversed phase resolving C18. The peptide trap was mounted on the loop of a Dionex Ultimate 3000 nano series (Thermo Scientific, USA). Following a wash with 0.1% formic acid for 30 min at 0.5 μL/min, the efflux of the peptide trap column was directed to a 10 cm resolving reversed-phase column (100 Å, 5 μm Magic C18 particles, Michrom Bioresources), which was mounted on the electrospray stage of a Velos Pro mass spectrometer (LTQ, Thermo Scientific). The peptides was separated on-line using 15 salt steps (0, 10, 30, 50, 100,150, 200, 250, 300, 350, 400, 500, 1,000, 1,500, and 2,000 mM. NH_4_CH_3_OO) followed by a 0–35% acetonitrile gradient for 120 min at a flow rate of 350 nL/min. An electrospray voltage of 1.9 kV was used, with the ion transfer temperature set to 250°C. The mass spectrometer was controlled by the Xcalibur software, which continuously performed mass-scan analysis of the LTQ and, subsequently, of the six most intense ions during MS/MS scans of the ion traps. For this, one repeat scan of the same ion was dynamically excluded, using a 30 s repeat duration and 90 s exclusion duration. Normalized collision energy for the MS/MS was set to 35%.

### Data Analysis Using Database Search Algorithm

All tandem mass spectra MS/MS samples were analyzed using SEQUEST (v1.4.0.288; Thermo Fisher Scientific, San Jose, CA, USA) and X! Tandem (vCYCLONE 2010.12.01.1; The GPM, thegpm.org). SEQUEST searched the National Center for Biotechnology Information (NCBI) *Piscirickettsia salmonis* 12-21-2015.fasta database (10,012 entries) assuming digestion of the enzyme trypsin. X! Tandem searched a subset of the *Piscirickettsia salmonis* NCBI 11-03-2016 database, also assuming trypsin digestion. SEQUEST and X! Tandem were searched with a fragment ion mass tolerance of 0.80 Da and a parent ion tolerance of 2.5 Da. Carbamidomethyl-cysteine was a fixed modification in SEQUEST and X! Tandem. In SEQUEST, asparagine and glutamine deamidation and methionine oxidation were variable modifications. In X! Tandem, Glu->pyro-Glu of the N-terminus, ammonia-loss of the n-terminus, gln->pyro-Glu of the N-terminus, asparagine and glutamine deamidation, and methionine oxidation were variable modifications.

### Criteria for Protein Identification

Scaffold (v.4.5.0; Proteome Software Inc., Portland, OR, USA) was used to validate MS/MS-based peptide and protein identifications. Peptide identifications were accepted if the Peptide Prophet algorithm, with Scaffold delta-mass correction established a > 95.0% probability (30). Protein identifications were accepted if presenting a > 99.9% probability as assigned by the Protein Prophet algorithm, and containing at least two identified peptides (31). Proteins containing similar peptides that could not be differentiated based on MS/MS analysis alone were grouped.

### Statistical Analysis

Assumptions of both variance normality and homogeneity were tested. For each vaccine variable, one- or two-way ANOVA general linear models were used. A two-way ANOVA was performed for each immune variable, with the factors being vaccinated fish and time. A Tukey's *post-hoc* test identified significantly different groups (α = 0.05).

## Results

### Identification of *Piscirickettsia salmonis* Protein Profiles for Vaccine Candidates

A previously developed liquid medium for cultivating large amounts of *P. salmonis* (10) was used to mainly culture the AUSTRAL-005 strain. From this, and according to what was described in materials and methods (Production of *Piscirickettsia salmonis* protein), the different protein fractions corresponding to each vaccine prototype (P1, P2, and P3) were visualized by SDS-PAGE (Figure 2). Where later prototype 1 was characterized with more than 86 proteins, where some have high immunogenic capacity (7, 10, 17, 18, 21). Which we were able to analyze by having in previous studies the draft of the genome sequence and the gene sequences of the virulent strain AUSTRAL-005 are known (32), and against *P. salmonis* vaccine prototypes were developed by using these data. Focus was given to proteins involved in host-pathogen interactions or to immunoreactive antigens secreted or located on the surface of other known pathogens (7, 32, 33). The protein fractions of the AUSTRAL-005 SRS strain, which is very infectious and produces a high cytopathic effect (24, 34), due to this the existence of different proteins is suggested which can be observed clear differences in the electrophoretic protein profile del prototype 1 with respect to prototype 2 and prototype 3 (Figure 2).

### Prototypes Vaccine Against *Piscirickettsia salmonis* Induces Innate Immune Responses in Atlantic Salmon

**Figure 3:**
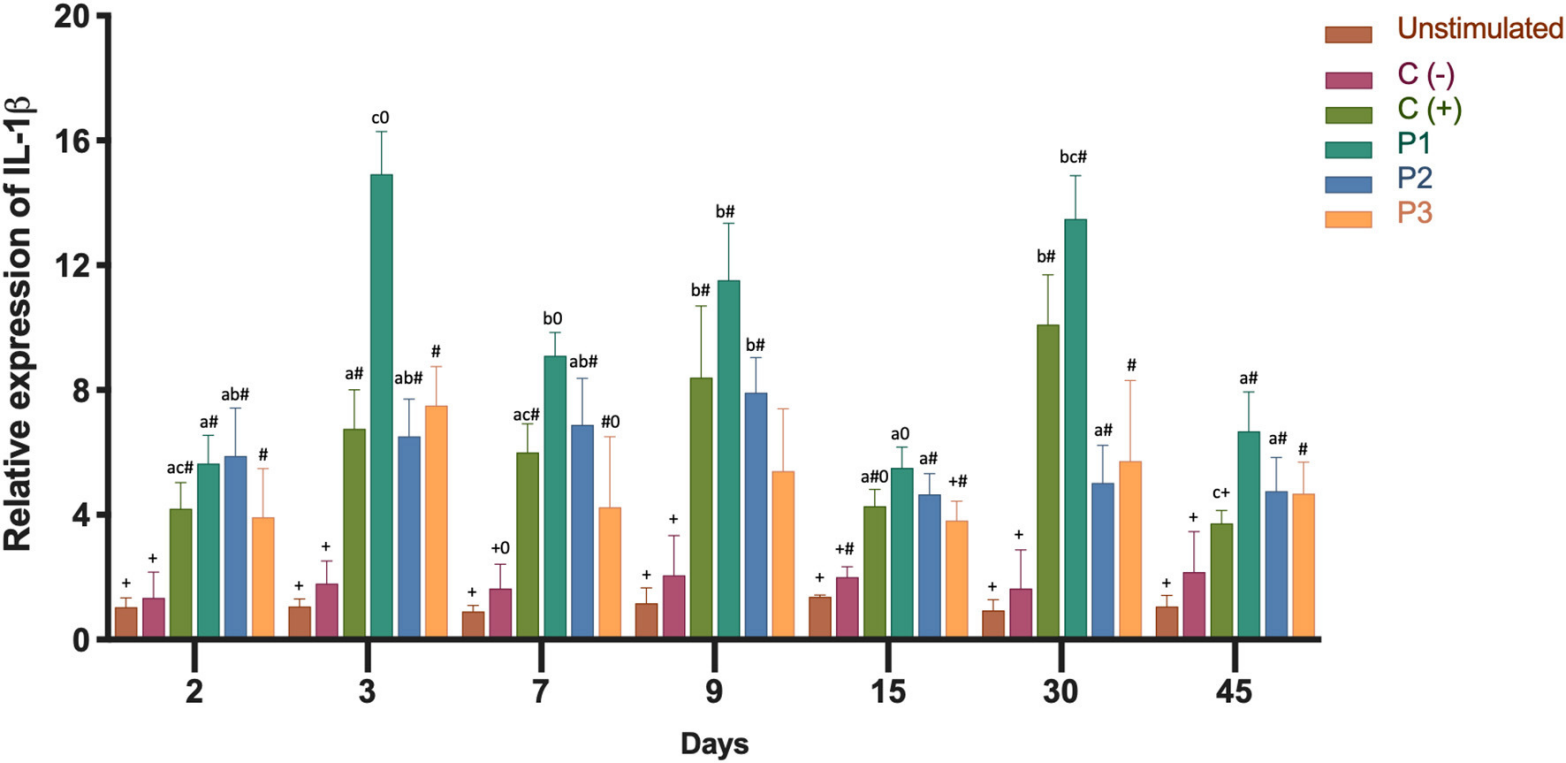
Relative expression of IL-1β mRNA transcription levels in head kidney 2, 3, 7, 9, 15, 30, 45 days after immunization. Fish from each treatment group were sampled at each time point. The mRNA level was measured by the means of real time qPCR, and the mRNA level in the stimulated cells was related to the mRNA level in control cells of unvaccinated fish. Prototype 1 (P1), Prototype 2 (P2), and Prototype 3 (P3); Positive controls (C+) commercial *P. salmonis* vaccine, and negative controls (C-) used PBS mixed with a non-mineral oil adjuvant. Values are mean + SEM. Notations above the bars indicate significant differences among days of experiment. Symbols indicate significant differences between the control and infested fish group (nested ANOVA, *post-hoc* Tukey-Test, *P* < 0.05).

**Figure 4:**
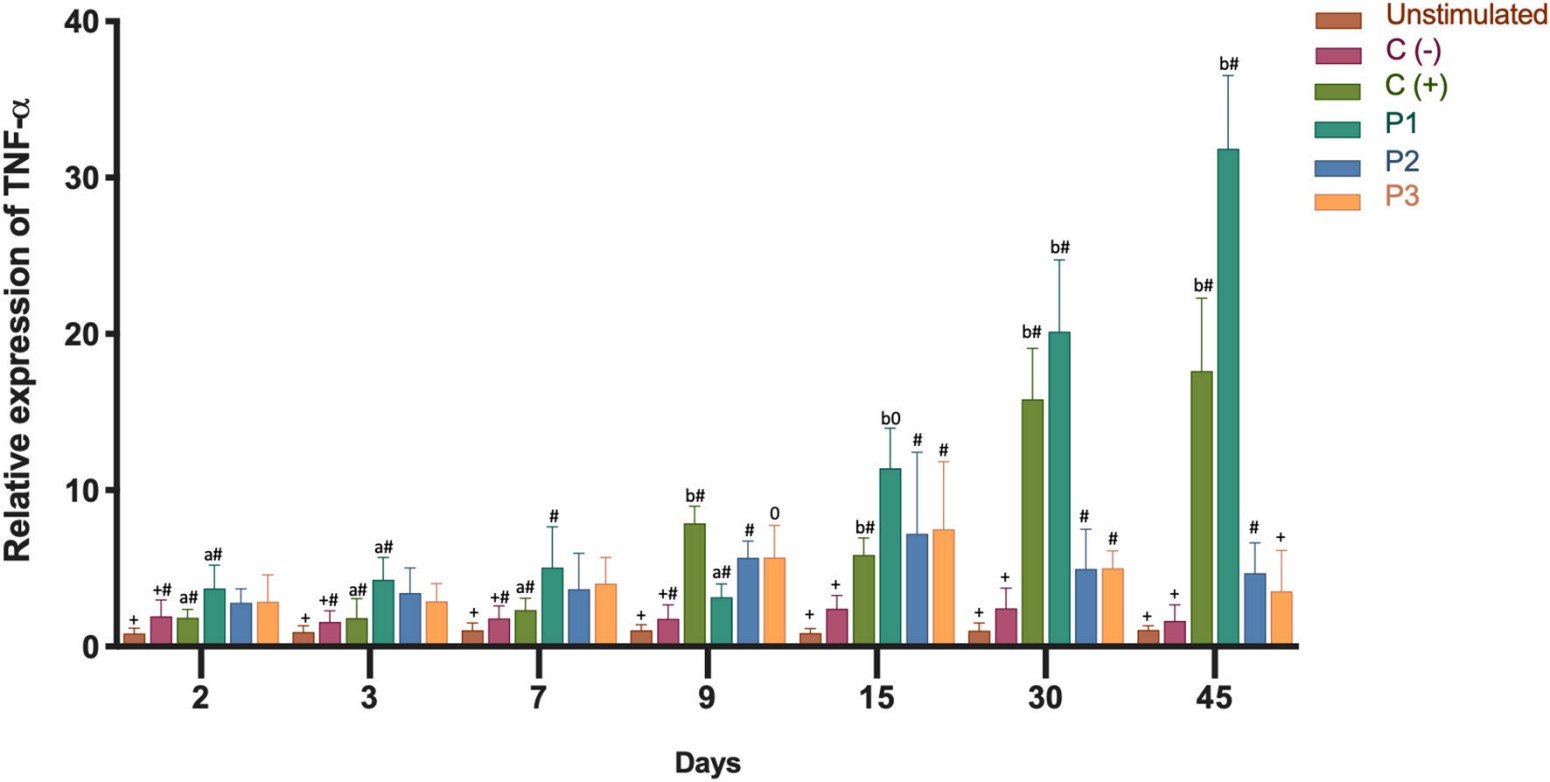
Relative expression of TNF-α mRNA transcription levels in head kidney 2, 3, 7, 9, 15, 30, 45 days after immunization. Fish from each treatment group were sampled at each time point. The mRNA level was measured by the means of real time qPCR, and the mRNA level in the stimulated cells was related to the mRNA level in control cells unvaccinated fish. Prototype 1 (P1), Prototype 2 (P2), and Prototype 3 (P3); Positive controls (C+) commercial *P. salmonis* vaccine, and negative controls (C-) used PBS mixed with a non-mineral oil adjuvant. Values are mean + SEM. Notations above the bars indicate significant differences among days of experiment. Symbols indicate significant differences between the control and infested fish group (nested ANOVA, *post-hoc* Tukey-Test, *P* < 0.05).

In order to determine the inflammatory immune response, we analyzed the expression of the transcripts of IL-1β and TNF-α. Both cytokines showed high gene expression in the head kidney as a result of immunization (Figures 3, 4). Three groups of fish were injected intraperitoneally with each *P. salmonis* protein-fraction vaccine prototype (i.e., P1, P2, and P3). These groups were compared to a commercial *P. salmonis* vaccine (C+) and negative controls [C- (PBS immunized plus adjuvant)], previously described. Non-injected fish were used as an unstimulated control. Head kidney samples for each group were analyzed at 2, 3, 7, 9, 15, 30, and 45 days. A significant increase (*p* < 0.05) in IL-1β transcript was detected 3, 9, 30, and 45 days after P1 vaccination as compared to the other formulations (P2 and P3) (Figure 3). Real-time qPCR data, meant to detected *P. salmonis* DNA over time (35), showed an absence of *P. salmonis* genome for 2–45 days within the bioassay samples (data not shown). Evaluation of TNF-α mRNA expression showed significant changes 30 and 45 days after vaccination with the prototype P1 v/s the negative control (C-) (*P* > 0.05) (Figure 4). These results suggest that administration of the P1 against *P. salmonis* vaccine is able to induce an innate immune response early in mRNA expression for IL-1β and, far later, for increased TNF-α transcript in comparison with the other two formulations.

### One of the Prototype Vaccines Effectively Protects Against *Piscirickettsia salmonis* Challenge

**Figure 5:**
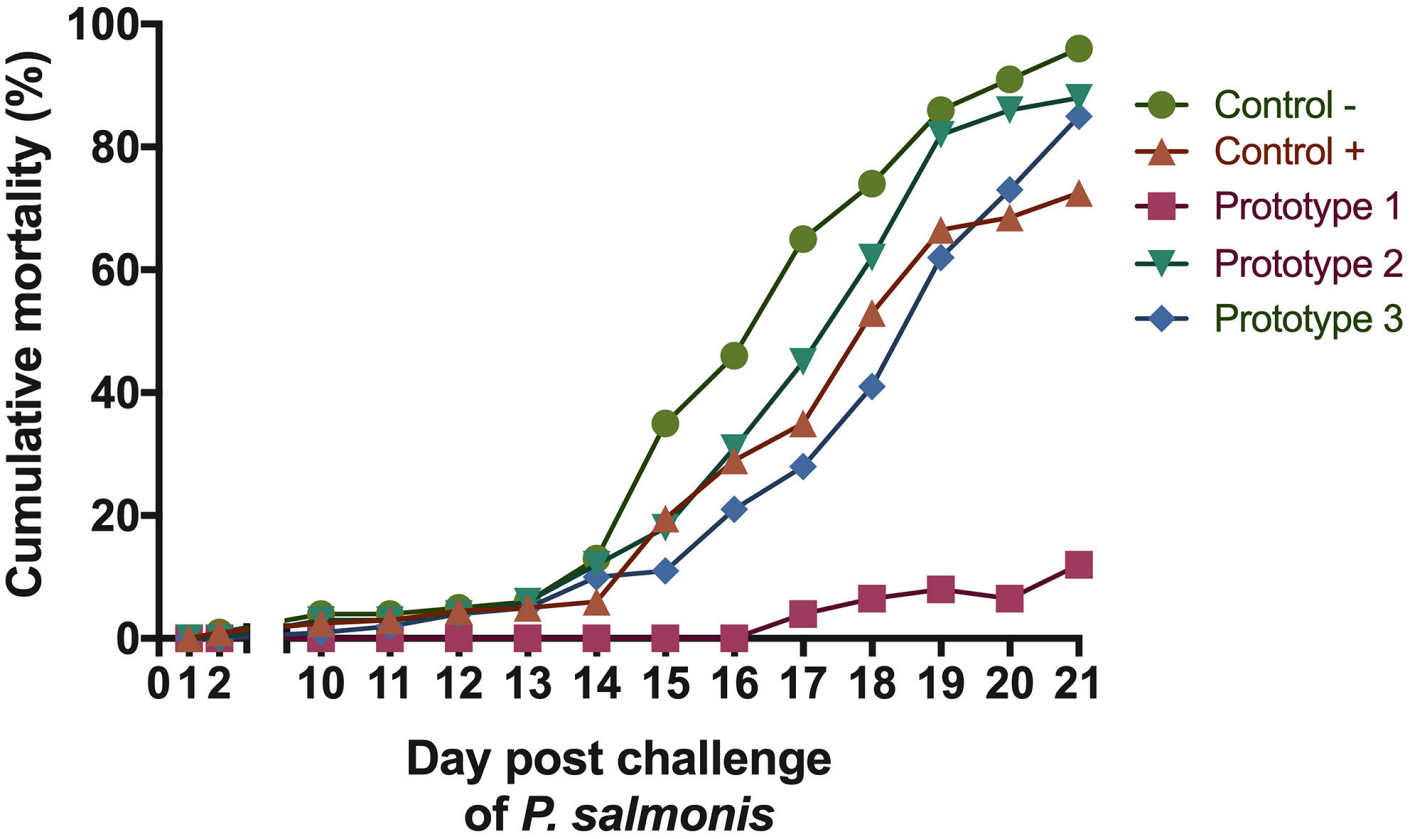
Challenge experiment with *P. salmonis*. Percentage cumulative mortality of *S. salar* vaccinated with the three formulations evaluated in the bioassay, post-ip challenge with *P. salmonis*.

**Figure 6:**
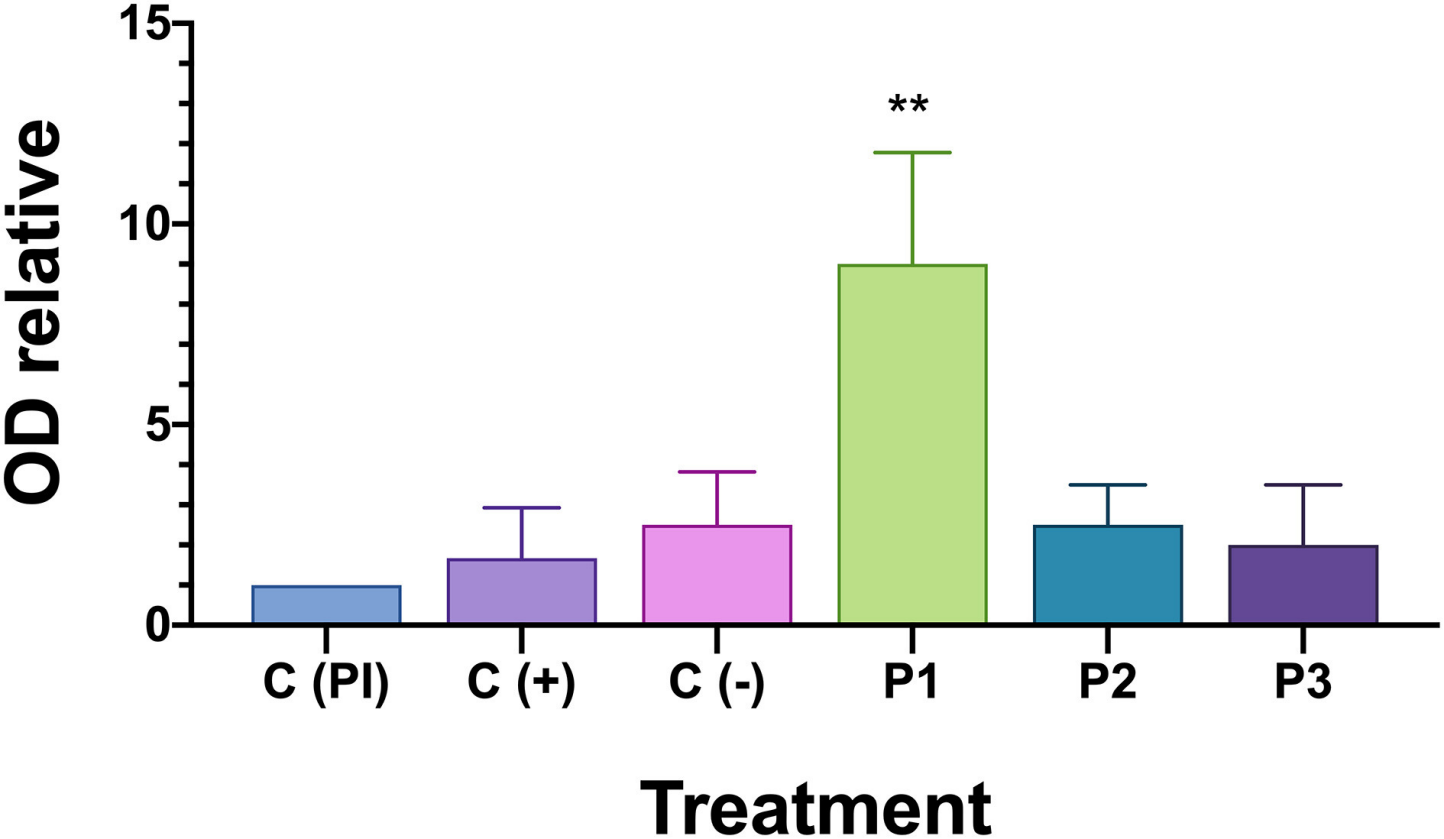
IgM in serum of fish challenged with *P. salmonis* by indirect ELISA. OD relative is measured optical density (OD) at 450 nm for each fish is divided on the optical density of the positive pre-immune control [C(PI)] on each plate. Asterisk indicates statistical difference by one-way ANOVA (*P* < 0.05).

A large majority of fish in the PBS-injected control group died 21 days after the introduction of *P. salmonis*. Septicaemia symptoms was observed in the liver or (head and posterior) kidney of diseased fish following injection and the infection was confirmed by PCR (25) (data not shown). To evaluate the protective capacity of the three prototype vaccines, fish were given lethal intraperitoneal injections of *P. salmonis* (Austral-005 strain), and daily fish mortalities were monitored (Figure 5). The relative percent survival (RPS) of the P1 vaccinated fish (89.6%) was higher than P3 (11.46%) and P2 (8.33%) vaccinated fish, as well as the positive control (C+, 26.1%) 21 days after exposure. These results directly correlated with the recorded specific IgM response (Figure 6). The prototype P1 increased both innate and specific adaptive immunity through the expression of pro-inflammatory cytokines and a strong *P. salmonis* specific IgM- response. These data strongly suggest that the prototype P1 effectively induces antibodies production that can protect against this pathogen (Figures 5, 6).

### Prototype P1 of Vaccine Induces Immunity in Fish Vaccinated and Challenged With *Piscirickettsia salmonis*

Specific IgM anti-*P. salmonis* production in the serum of surviving fish was evaluated to assess if the vaccine induces against bacteria specific antibodies after Austral-005 strain exposure. Anti-*P. salmonis* antibody were detected using an indirect ELISA assay (Figure 6). Only vaccine prototype P1 induced a stronger immune response through the generation of antibody titers in the serum 21 days after the *P. salmonis* challenge. These results could suggest acquired immunity since *S. salar* responded faster and more accurately in generating IgM against *P. salmonis* antigens, suggesting immunological memory is necessary to activated in the case of this intracellular bacteria.

### Proteome Analysis of the *Piscirickettsia salmonis* Protein-Fraction Vaccine Prototype 1

**Figure 7:**
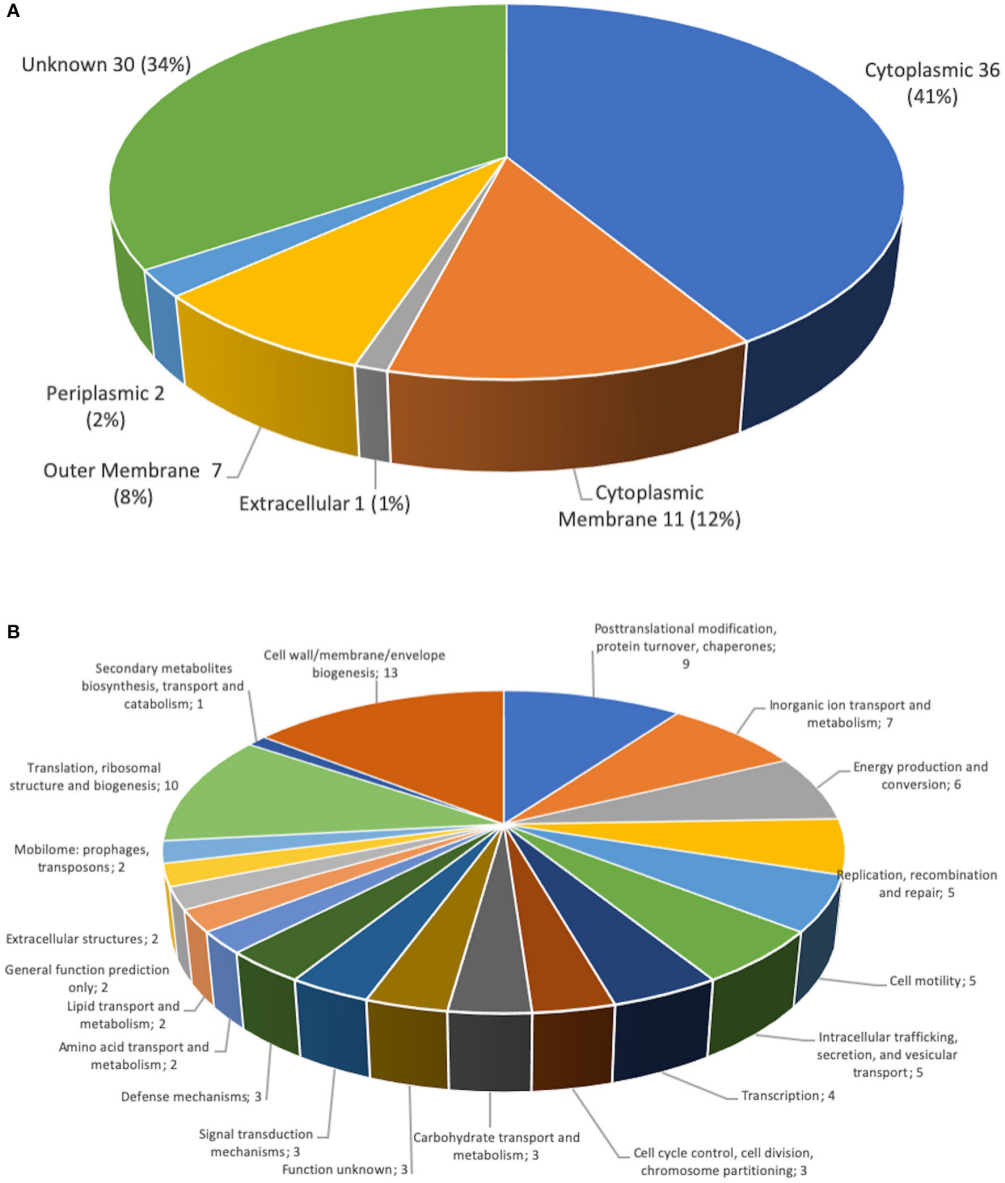
Classification of *P. salmonis* 005-Austral SRS total fraction proteins. **(A)** Subcellular locations of total fraction proteins identified by MudPIT. Predicted subcellular locations of the 87 total fraction proteins identified using PSORT3b. **(B)** Functional classification of *P. salmonis* 005-Austral SRS total fraction proteins. The 87 proteins identified by MudPIT were sorted according to the indicated clusters of orthologous groups.

In order to identify the proteins components the proteome of the prototype 1, was performed. The purified protein of prototype 1 from *P. salmonis* Austral-005 showed a total of 87 unique associated proteins were identified. The 28 most-abundant proteins from the purified of the protein fraction are listed in Table 1. The subcellular localization of the proteins identified, classified by the PSORTb v.3.0 program (36). This classification was organized into six groups: (1) cytoplasmic membrane proteins, (2) cytoplasmic proteins, (3) periplasmic proteins, (4) outer membrane proteins, (5) extracellular proteins, (6) proteins of unknown location. Of these 87 proteins identified from the total fraction of *P. salmonis*, 36 (41%) were cytoplasmic, 11 (12%) of the cytoplasmic membrane, 7 (8%) of the outer membrane, 2 (2%) of the periplasm, 1 (1%) extracellular, and 30 (34%) were of unknown location (Figure 7A). The composition of this protein localization of the *P. salmonis* protein-fraction vaccine prototype1 was mainly cytoplasmic and cytoplasmic membrane, therefore a varied composition of various compartments in the formulation of the SRS vaccine is observed.

**Table 1:** Classification of virulence-related proteins identified from total fraction proteins to *Piscirickettsia salmonis*.

**Classification**	**Accession number NCBI**	**VFDB Gene name**	**VFDB Description**
Adherence	ERL63261	*cadF*	Fibronectin-binding protein
	WP_052104618	*pilA*	Type IV pilin
	ALA23777	*pilC*	Type IV pilus biogenesis protein PilC
	ALB21306	*cadF*	Outer membrane fibronectin-binding protein
	WP_036771893	*omp89*	Outer membrane protein
	ALB23929	*pilJ*	Twitching motility protein PilJ
	WP_016210084	*IlpA*	Immunogenic lipoprotein A
Efflux pump	WP_016209619	*adeF*	RND efflux transporter
	ERL61562	*nrpX*	MFS family transporter
Elongation factor	WP_016209251	*tuf*	Elongation factor Tu
	WP_032126260	*tuf*	Elongation factor Tu
Enzyme	WP_032126147	*katA*	Catalase
	WP_017376766	*mip*	Macrophage infectivity potentiator
Flagellar	WP_016210447	*fliE*	Flagellar hook-basal body complex protein
Iron metabolism	WP_016209255	*fur*	Transcriptional repressor of iron-responsive genes (Fur family)
	WP_032126547	*hasF*	Outer membrane channel protein
Secretion system	ALA25850	*icmG/dotF*	Dot/Icm type IV secretion system core complex protein IcmG/DotF
	WP_017378270	*icmE/dotG*	Dot/Icm type IV secretion system core complex protein IcmE/DotG
	AOS36969	*trwE*	TrwE protein
	WP_016210039	*ssaN*	Type III secretion system ATPase
	WP_016209722	*virB9-1*	Type IV secretion system protein VirB9
Stress protein	KLV35114	*sodCI*	Gifsy-2 prophage: superoxide dismutase precursor (Cu-Zn)
	ALA24403	*ahpC*	Alkyl hydroperoxide reductase subunit C, AhpC (alkyl hydroperoxidase C)
Other		*hasF*	Outer membrane channel protein
	KLV35288	*eno*	Enolase, putative
	WP_016210800	*bcfH*	Thiol-disulfide isomerase
	WP_016209655	*ML1683*	Histone-like protein
	WP_016209645	*tig/ropA*	Trigger factor

*The table contain the protein classification, accession number of NCBI Not Redundant database, VFDB gene name and VFDB description. The Virulence Factor data were extracted from the Virulence Factor Database*.

A functional classification was made of the proteins identified in the proteome of the *P. salmonis* protein-fraction vaccine prototype 1 according to the orthologs groups (COG). Where they were identified in the cell wall, membrane and envelope (13 proteins). In addition, nine proteins in post-transcriptional modification, protein turnover, chaperones, seven proteins in transport and metabolism of inorganic ions, six proteins in production and conversion of energy, five proteins in replication, five protein in cell motility, five proteins in intracellular traffic, four proteins transcription, three proteins in the control of the cell cycle, three proteins in carbohydrate transport, three proteins in signal transduction mechanism, three proteins in defense mechanism, 10 proteins in translation, two proteins in mobilome, two proteins in extracellular structures (Figure 7B). The identification of diverse functions but mainly in the biogenesis of the cell wall, the membrane and the proteins associated with post-transitional modifications and chaperones.

### Proteins Associated With Virulence Contained in the *Piscirickettsia salmonis* Protein-Fraction Vaccine Prototype 1

The annotations and classification regarding the virulence factors of the most abundant proteins of the *P. salmonis* protein-fraction vaccine prototype 1, was performed using the DIAMOND software (37), considering the cutoff of 1e-10. The analysis showed that 28 proteins of the total fraction, have a high prognostics of bacterial virulence, of these were associated with adherence as: *cad*F, *pil*A, *pil*C, *pil*J, *IlP*a, *omp*89, besides being some of them associated with biogenesis of pilus type IV (38, 39). In addition proteins were found associated with efflux pump as *ade*F, *nrp*X. Important also are the proteins associated with the conformation of the flagellar motor as it is *fli*E, which has been described previously as a structural part of the flagellar motor, however, transcriptomic analyzes performed under different bacterial culture conditions, the genetic non-expression of *fli*E has been determined (40), which will suggest that the characteristics in the protein expression of the AUSTRAL-005 strain they are different and which can lead to an expression of virulence factors, and addition to the proteins associated with different secretion systems such as: *icm*G/*dot*F and *vir*B9-1 (secretion system type IV), ssaN (type III secretion system), and various external membrane proteins such as: *has*F, *cad*F, and *amp*89, among other proteins (Table 1). Also proteins involved in the regulation of iron uptake (fur), which has been described as protein modulates the genes involved in iron uptake, such as those related to siderophore biosynthesis, receptors and transporters. This transcription factor also limits excess iron entry into the bacterium (41).

## Discussion

Internationally, infectious diseases are a serious factor affecting aquaculture development. In Chile, SRS is considered the main cause of mortality among cultured salmonids (17) Unfortunately, antibiotic use has been unsuccessful in controlling outbreaks. The need for antibiotic treatments could be significantly reduced through the use of vaccination, which could effectively prevent infectious diseases. Although vaccines against bacterial diseases have great potential in aquaculture, vaccine development is extremely complex (42). More than 33 vaccines exist in the Chilean market against SRS (17), but these have had variable results and poorly documented efficacies (26, 43, 44). Current vaccine development against *P. salmonis* is hindered by the various virulence factors and pathogenic mechanisms presented by this bacterium. Since transcription could be the key for fully understanding the host-pathogen interaction, the current report developed the P1 vaccine prototype based on the AUSTRAL-005 strain due to an availability of genomic data and transcriptome analyses (24). For this reason, in this work we try to perform a complete analysis with different omics tools such as the proteomic analysis of the protein fraction (P1, vaccine prototype 1) that offers greater protection against the pathogen during field trials, in order to understand the effectiveness of this vaccine against *P. salmonis*. The developed vaccine included highly expressed major antigenic proteins, with results supporting the administration of microbial antigens as immunocomplexes, which improved innate and acquired immune responses to a highly pathogenic *P. salmonis* strain (32, 45).

The innate immune response, a central fish defense mechanism, is important for activating an acquired immune response (46). Previous studies have demonstrated the feasibility of stimulating an acquired immune response to *P. salmonis* antigens (7, 9). Nevertheless, these studies focus on evaluating antibody levels and RPS after a challenge, without evaluating specific immune responses. This lack of evaluation has left open for debate the antigens to best stimulate the acquired immune system, as well as the exact nature of their effect (47). This study developed a *S. salar* immunization treatment tested via a challenge with *P. salmonis* that included evaluations of various innate immunity parameters, including the expression of transcripts encoding major cytokines (IL-1β and TNF-α). Furthermore, this study evaluated the effect of immune complexes on the acquired humoral immune response, allowing comparisons to be made with the antibody levels in challenged fish.

Cytokines have crucial roles in regulating the immune response (48) and in mediating the effector phase of both innate and adaptive immunity (49). One key, early response, pleiotropic cytokine is IL-1β, which is secreted when pathogens enter circulation. The IL-1β receptor is expressed in all Atlantic salmon tissues (50). One function of Il-1β is to stimulate the vascular endothelium and secrete IL-6, thereby initiating protein synthesis and the acute phase response (51). IL-1β was found highly expressed in the 1st days after immunization, with significant expression in fish injected with the P1 vaccine (Figure 3). The P2 and P3 vaccines also increased IL-1β expression when compared to the control. TNF-α, secreted by leukocytes another pleiotropic cytokine, exerts a pro-inflammatory effector mechanism. This cytokine acts as an important factor in the activation of macrophages, resulting in respiratory bursts and phagocytosis (52). In rainbow trout head kidney leucocytes, TNF-α increase phagocytosis and chemotaxis to induce IL-1β and IL-8 expression (48). TNF-α transcript expression was found to be significantly up-regulated in the present study, especially when fish were stimulated with the P1 vaccine. The greatest expression was achieved in the final days of the test period (Figure 4). Analyses of IL-1β and TNF-α mRNA expressions showed significant stimulation of the specific immune response by the P1 formulation in comparison with the stimulation observed by P2 and P3 prototypes (Figures 3, 4). *In vivo* measurements of IL-1β and TNF-α mRNA expressions in the bioassay correlated with previous *in vitro* studies that showed increases in both cytokines after cell incubation with SHK-1 protein-based vaccine prototypes (1, 2, and 3) of *P. salmonis* (53).

Three months after vaccination, fish were challenged with 10^8, 5^ TCID_50_/mL formulations of *P. salmonis*. The cumulative mortality of negative control (C-) fish was 96% (Figure 5), supporting the high pathogenicity of the *P. salmonis* strain used, where in addition we obtained protection with a relative survival percentage of (RPS) 89.6% as compared to the control group, which also demonstrated high antibody titers against both proteins in the inoculated salmon serum 3 months after vaccination.

Three different immunoglobulin (Ig) isotypes can be found in teleost fish, IgM, IgD, and the teleost-specific IgT. IgM is considered to have a systemic activity, and IgT is attributed a mucosal role, similar to mammalian IgA. In most teleostean species, the basal expression of *IgM* is dominant, followed by *IgT*. The highest levels of Ig expression are in head kidney, generally followed by spleen (22, 54). Regarding disease prevention and control,

IgM is an important immunoglobulin class for the fish farming industry (55). IgM is very important in phylogenetic research since it is often the only class of immunoglobulin described in fish, in addition to being important in an ontogenetic context as the largest primary antibody among higher vertebrates (56), for effective vaccination against *P. salmonis*, it must be based on the ability to stimulate adaptive immunity and long-term memory responses. This study examined the levels of serum IgM in fish surviving a challenge with *P. salmonis*. A specific antibody titer was found, suggesting that increased antibody titers correlated with post-challenge survival rates. Challenging vaccinated fish with a particular pathogen appears to be an effective direct method for evaluating vaccine potential (Figure 6).

In relation to the differential origins, the proteins identified in prototype 1 of the *P. salmonis* vaccine presented various functions. By means of the COG definition, cell wall, membrane and envelope (13 proteins) were classified (Figure 7). In addition, nine proteins in posttranscriptional modification, protein turnover, chaperones, seven proteins in transport and metabolism of inorganic ions, six proteins in production and conversion of energy, five proteins in replication, five protein in cell motility, five proteins in intracellular traffic, four proteins Transcription, three proteins in the control of the cell cycle, three proteins in carbohydrate transport, three proteins in signal transduction mechanism, three proteins in defense mechanism, 10 proteins in translation, two proteins in mobilome, two proteins in extracellular structures, some proteins such as: VirB9, VirB10, TraF, IcmG / DotF are required as a component of the type IV secretion system (57), which has been shown to induce humoral and immunity (38). Also associated were proteins that are related peptidoglycan-associated (lipo) proteins and Outer membrane protein as OmpA, Omp89, TolC, in addition to ABC-type metal ion transport components, and periplasmatic component. Several proteins related to antibiotic resistance have been identified in this proteome analyzed by our group, where they include: TolC, AcrA, MFS, and members of multidrug effusion pumps type RND (58). Additionally, several proteins involved transcriptional repressor of iron-responsive genes (Fur family) were identified (41). In turn, a protein wrapped in Flagellar hook-basal body complex protein has been found, such as FliE, which had not previously been described its genetic expression in other culture conditions (40). Within the sequenced proteome, we have been able to identify different proteins classified with adhesion properties such as: cadF, pilA, pilC, pilJ, omp89, IlpA (Table 1), where they belong to the type IV pilus is a filamentous structure existent on the surface of various pathogenic bacteria, such as *Legionella pneumophila* (59), *Pseudomonas aeruginosa* (60), and the fish pathogens *Aeromonas salmonicida* subsp. *Salmonicida* (61), *Vibrio anguillarum* (62), and *Piscirickettsa salmonis* type strain LF-89 (39). Which makes us presume that this first contact that the bacteria should have with the host is of great importance to trigger the immunogenic response and in this same way they are excellent candidates to consider for the formulation of future therapies.

The high amount of proteins in the prototype 1 vaccine involved in the survival of the pathogen, suggests that these mechanisms have a high immunogenicity rate of enveloped proteins, which causes greater protection than the other vaccine prototypes. We sought to divide the analysis in two main mechanisms that the bacteria could use to interact with the host. In particular, we analyzed virulence factors such as adherence, efflux pump, elongation factor, enzyme, flagellar, iron metabolism secretion system stress protein, as described previously.

In conclusion, our results show that all three-vaccine prototypes stimulated the innate immune system by increasing the transcript expression of IL-1β and TNF-α, two highly important marker cytokines. These data suggest that there is a correlation between activation of the innate immune system and protection against mortality. Vaccination with the P1 prototype resulted in an elevated survival rate when fish were challenged with *P. salmonis*, and this strongly correlated with a high induction of IgM specific against *P. salmonis*. The exact protective mechanism of the induced IgM antibodies against *P. salmonis* must be studied to understand the role of this immunoglobulin during the early infection stages and secondary multiplication in different tissues. Related studies by our group (34) demonstrate that IgM against the P1 prototype can inhibit bacterial growth, suggesting that circulating anti-*P. salmonis* antibodies may limit the growth and spread of bacteria in fish organs. The present study identified 87 proteins in the prototype 1 of *P. salmonis*, which is an important contribution to the way of generating highly efficient vaccines for control of SRS. Which is value information for future studies to combat this important pathogen that affects the salmon industry. Where we can determine that this vaccine does not produce immunosuppression at the concentrations used in this study, the opposite of what happens with other commercial bacterin formulations that are used today.

We suggest that this new vaccine formulation against *P. salmonis* be used to prevent your widespread infection. Furthermore, a boost may be used after primary vaccination to maintain increased levels of circulating protective antibodies. This protective effect is important in improving vaccine efficacy for full, long-term protection, which would ultimately reduce salmon industry losses caused by this pathogen.

## Data Availability Statement

The datasets presented in this study can be found in online repositories. The names of the repository/repositories and accession number(s) can be found at: https://www.ebi.ac.uk/pride/, PXD023109.

## Ethics Statement

The animal study was reviewed and approved by Foundation of Chile and bioethical committees of the Universidad Austral de Chile and the National Commission for Scientific and Technological Research (ANID) of the Chilean government.

## Author Contributions

JP conceived of and designed the study and drafted the manuscript. CE conceived of and designed the manuscript. GN performed the proteomic analysis. CO performed the protein fractionation. RA-H, JF, and CR contributed in the draft of the manuscript. JT contributed to the experimental design. LV-C contributed in the initial draft of the manuscript and statistical analysis. AY contributed in the initial draft of the manuscript and experimental design. All authors contributed to the article and approved the submitted version.

## Conflict of Interest

The authors declare that the research was conducted in the absence of any commercial or financial relationships that could be construed as a potential conflict of interest.
